# Measuring quality of gout management in residential aged care facilities

**DOI:** 10.1093/rap/rkac091

**Published:** 2022-11-28

**Authors:** Amy D Nguyen, Kimberly E Lind, Richard O Day, Daniel Ross, Magdalena Z Raban, Andrew Georgiou, Johanna I Westbrook

**Affiliations:** Centre for Health Systems and Safety Research, Australian Institute of Health Innovation, Macquarie University, Sydney, NSW, Australia; St Vincent’s Clinical School, UNSW Medicine, UNSW Sydney, Sydney, NSW, Australia; Centre for Health Systems and Safety Research, Australian Institute of Health Innovation, Macquarie University, Sydney, NSW, Australia; St Vincent’s Clinical School, UNSW Medicine, UNSW Sydney, Sydney, NSW, Australia; Department of Clinical Pharmacology & Toxicology, St Vincent’s Hospital, Sydney, NSW, Australia; Faculty of Medicine, Health and Human Sciences, Macquarie University, Sydney, NSW, Australia; Centre for Health Systems and Safety Research, Australian Institute of Health Innovation, Macquarie University, Sydney, NSW, Australia; Centre for Health Systems and Safety Research, Australian Institute of Health Innovation, Macquarie University, Sydney, NSW, Australia; Centre for Health Systems and Safety Research, Australian Institute of Health Innovation, Macquarie University, Sydney, NSW, Australia

**Keywords:** gout, long-term care, co-morbidity, homes for the aged, guideline concordance

## Abstract

**Objective:**

Gout, a common form of arthritis, can be controlled successfully with pharmacotherapy and is thus an ideal model for examining chronic disease management. Our aim was to examine treatment of gout evaluated in accordance with general management guidelines for gout as applied to Australian residential aged care facilities.

**Methods:**

Electronic health record data linked with aged care clinical notes and electronic medication administration information (11 548 residents in 68 residential aged care facilities, >65 years of age) were interrogated to identify people with gout, other chronic conditions and gout medication use. The outcomes examined were the proportion receiving urate-lowering therapy (ULT; preventative medication) and/or colchicine/non-steroidal anti-inflammatory drug (NSAID) (to treat gout flares), the number of ULT and colchicine/NSAID treatment episodes (periods of continuous days of medication use) and the duration of these treatment episodes.

**Results:**

The cohort included 1179 residents with gout, of whom 62% used a ULT, with a median of one episode of use for a very short duration [median = 4 days, median of use in total (i.e. repeated use) = 52 days]. Among residents with gout, 9% also used colchicine or an NSAID. Female residents were less likely to receive ULT and for shorter periods.

**Conclusion:**

Nearly one-third of residents with gout did not receive ULT. In those receiving ULT, recurrent short courses were common. Overall, management of gout in aged care residents appears to be suboptimal, largely owing to intermittent and short exposure to ULT, and with female residents at greater risk of poor gout management.

Key messages

- Interrogation of electronic medication administration data from residential aged care facilities identified suboptimal management of gout within this setting.
- One-third of residents did not receive preventative urate-lowering therapies or received them in guideline non-concordant ways, and only 9% used colchicine/NSAID for gout flares.
- Sex differences in gout management of residents existed, with female residents being less likely to receive urate-lowering therapies and for shorter periods.

## Introduction

Gout is a chronic form of inflammatory arthritis that occurs in individuals with elevated concentrations of serum urate. Acute gout is characterized by episodes of sudden, often very severe pain in the affected joints, termed gout flares. Chronic gout is characterized by recurrent gout flares. If left untreated, gout can lead to deposits of monosodium urate crystals permanently damaging and deforming joints. Subcutaneous nodules, typically located over joints, called tophi, also damage joints but also lead to kidney damage [1]. The pain and subsequent loss of mobility that gout causes can lead to social, emotional, occupational and financial impacts that have been underrated [2, 3].

People with gout commonly have co-morbidities, and this is more likely in elderly people with gout who, consequentially, experience reduced quality of life [4, 5]. The medications required to manage these co-morbid conditions can result in polypharmacy, increasing the likelihood of deleterious drug–drug interactions involving gout treatments [4]. Gout is relatively common among residents in residential aged care facilities (RACFs; also known as nursing homes or long-term care facilities) [6]. These residents are also likely to have multiple conditions, such as hypertension, heart disease, diabetes and a history of myocardial infarction [6].

Australian guidelines for the effective management of gout (summarized in Supplementary Table S1, available at *Rheumatology Advances in Practice* online) are similar to multinational guidelines, such as the 3e initiative [7] and national guidelines used in the UK [8], USA [9, 10] and Europe [11]. All guidelines recommend the use of urate-lowering therapies (ULTs), such as allopurinol, as the mainstay treatment to prevent gout flares and long-term consequences of poorly controlled gout. Allopurinol effectively reduces serum urate concentrations in those with chronic gout [12]. However, many studies have shown that ULTs are often not prescribed when they are indicated [13, 14] or do not achieve serum urate targets associated with successful control of gout owing to inadequate dosing [12]. Furthermore, gout flares can be treated with unsafe regimens in older people, such as excessive doses of colchicine in renal impairment, prolonged dosing with glucocorticosteroids (e.g. prednisolone) and use of NSAIDs in people at high risk of gastrointestinal bleeding or heart failure [15]. Putting gout guidelines into practice in community settings has been difficult for a multitude of reasons [16]. Most gout is managed by general practitioners who feel that they lack awareness of guidelines about gout and its management [16]. Yet, general practitioners are responsible for managing the medical conditions of most aged care residents. Patients also have a poor understanding of the condition and its management [16].

There is increasing evidence that many conditions are not managed well in residential aged care, negatively impacting the quality of life of residents [17–19]. As the number of people using aged care services in Australia grows, and the age at admission and medical complexity increase, greater attention to the management of chronic health conditions is required [6, 20]. Given that most residents in RACFs receive their medications under supervision of carers and nurses, greater adherence to prescribed treatments compared with older adults in the community who are more likely to self-manage their medications, would be expected. In this study, we aimed to examine how gout management in RACFs aligns with current recommendations [21]. To achieve this aim, we took a new approach by using routinely collected electronic medical record data of residents in aged care facilities. Specifically, we examined the use of ULT and medications for gout flares in the gout cohort identified, with respect to resident length of stay (LOS), age, sex and co-morbidities.

## Methods

### Data

This dynamic retrospective cohort study used electronic health record data from 68 facilities from a large not-for-profit aged care provider with facilities across New South Wales and Australian Capital Territory, in Australia. From a sample of 11 548 people aged ≥65 years who were permanent residents (i.e. short-term respite care excluded) in these 68 facilities for ≥2 weeks during 2014–2017, we detected people who had gout. Diagnoses of gout and other chronic conditions were identified using information recorded anywhere in three parts of residents’ electronic health records: the Aged Care Funding Instrument (ACFI, which is form where the care needs of each resident are recorded to determine funding); free-text fields in the clinical notes for residents (these were searched for relevant text strings: gout, uric acid and tophi or tophus); and ULT (almost exclusively allopurinol) use recorded in medication administration records for residents. We have previously described this method of identifying health conditions in aged care residents [22]. The ACFI is an assessment of health and functional status used in Australia to determine the level of funding and services for which older people are eligible [23]. Analysis of text strings to identify health conditions has become possible only since the availability of electronic health records in RACFs.

Using medication administration records, we identified gout medications of interest: allopurinol, febuxostat, probenecid, colchicine and NSAIDs. These medications, with the exception of NSAIDs, are highly specific to gout treatment and have few other (and very rare) indications. Although NSAIDs are used for other musculoskeletal and arthritic conditions, we assumed gout to be the indication because we explored only those residents with a diagnosis of gout in their electronic health record and, as per guidelines, NSAIDs are used first line in patients with gout who have risk factors that do not permit colchicine use [24].

### Outcomes

We examined the management of residents with gout according to consolidated gout management guidelines (Supplementary Table S1, available at *Rheumatology Advances in Practice* online). We assessed ULT use, which is recommended in patients with chronic gout (defined as two or more gout flares in a 12-month period), and use of colchicine or NSAID, which should be used to treat gout flares. Glucocorticosteroids can also be used for this purpose but are used for a much wider variety of conditions other than flares of gout and are generally not considered first-line therapy [15], hence their use was not investigated in this study. We calculated the following primary outcomes: the proportion of residents with gout with any ULT use during the study period; the number of ULT use episodes; and the number of days of ULT use as a proportion of LOS. Colchicine/NSAID use was also explored. Thus, if a patient has had two gout flares within a year (colchicine/NSAID use is a proxy measure for gout flares) they should be on continuous ULT. Specifically, the following secondary outcomes were calculated: the number of colchicine or NSAID episodes initiated before ULT use; the number of colchicine or NSAID episodes initiated after ULT use ceased; the number of colchicine or NSAID episodes with concurrent ULT use; and the number of days of colchicine or NSAID use as a proportion of LOS. Medication episodes were defined as continuous days of use with 1-day gaps allowed (because gout ULT medications are required to be taken daily and long term) and with time away from the facility not counted as an interruption.

### Independent variables

Independent variables collected included age, sex, LOS, co-morbidities (hypertension, renal disease, dementia, diabetes and heart failure), facility remoteness (inner regional and outer regional, with major cities as the reference group). Independent variables were measured in the same manner as in our previous study that measured gout prevalence in an RACF cohort [6].

### Statistical methods

We also evaluated quadratic terms for age and LOS to allow curvilinear effects, and interactions between age and sex to allow age effects to vary by sex; we retained interactions and quadratic terms if they met the threshold of *P* ≤ 0.20 that indicated potential confounding [25]. We also controlled for left censoring (i.e. a resident using medication at admission who was assumed to be using ULT before admission) and right censoring (i.e. a resident using medication on the last day of stay or last day of the study period) when analysing the duration of medication use in relationship to LOS.

Descriptive statistics were calculated for all variables of interest. Generalized estimating equations regression was used to evaluate associations between the three primary medication outcomes and the independent variables, while accounting for correlation within facilities. For binary outcomes of any ULT use during the study period and ULT use as a proportion of LOS, we used a Probit-link function and binomial distribution. For the outcome of the number of ULT episodes, we used a gamma distribution and log-link function (selected using a modified Park test) [26].

From each model fit, we estimated marginal effects for all independent variables, which can be interpreted as the change in probability/proportion/days of use (depending on the outcome) associated with a change in the independent variable from the base/reference level.

### Ethics and consent

This research was approved by the University of New South Wales (HCI13091) and the Macquarie University Human Research Ethics Committees (5201401005). Individual consent was not sought because the study used retrospective electronic health record data. Permission for accessing data was granted through a Collaborative Research Agreement between the University of New South Wales and the aged care provider.

## Results

Of the 11 528 people in the cohort who met the inclusion criteria, 1179 (10%) had a diagnosis of gout. The characteristics of these residents are summarized in Table 1. The most common co-morbidities in residents with gout were hypertension (71%), dementia (47%) and diabetes (34%), and 49% of the gout cohort died during the study period.

**Table 1.** Sample characteristics of residents with gout between 2014 and 2017 from 68 residential aged care facilities

	Residents with gout (*n* = 1179)		
Variable	Frequency	Percentage	95% CI
Male	625	53.01	50.16, 55.85
English as primary language	1059	89.82	87.96, 91.43
Country of origin			
Australia	852	72.26	69.64, 74.75
UK	98	8.31	6.86, 10.03
China	15	1.27	0.75, 2.11
Italy	14	1.19	0.69, 2.00
Marital status			
Unknown	74	6.28	5.02, 7.82
Single	95	8.06	6.63, 9.76
Married	357	30.28	27.72, 32.96
Widowed	544	46.14	43.31, 48.99
Divorced	90	7.63	6.25, 9.30
Separated	19	1.61	1.02, 2.52
Co-morbidities			
Hypertension	842	71.42	68.77, 73.92
Dementia	550	46.65	43.82, 49.50
Diabetes	405	34.35	31.69, 37.11
Renal disease	334	28.33	25.83, 30.97
Myocardial infarction	82	6.96	5.63, 8.56
Died during study period	581	49.28	46.43, 52.13
Facility urban/rural category			
Major cities of Australia	831	70.48	67.82, 73.02
Inner regional Australia	313	26.55	24.11, 29.14
Outer regional Australia	35	2.97	2.13, 4.11
	**Median (interquartile range)**		
Age, years			
Female	87 (83–92)		
Male	85 (79–90)		
Length of stay, years			
Female	2.26 (0.87–4.64)		
Male	1.60 (0.62–3.56)		
Staff per bed	0.79 (0.60–1.05)		

Sixty-two per cent of residents with a diagnosis of gout in their electronic health record received ULT (Table 2), with a median of one ULT episode (range = 0–5). The median duration of a ULT episode was 4 days. The median total number of days when residents were on a ULT for the duration of the study, irrespective of gaps in treatments, was 52 days (range = 0–363).

**Table 2.** Urate-lowering therapy, colchicine and NSAID use in residents with gout (*n* = 1179)

Variable	*n*	Percentage
Gout patients on ULT	729	62
	Median	Interquartile range
Number of ULT episodes	1	0–5
Total days of ULT use	52	0–363
Length of ULT episodes, days	4	2–19
Time on ULT/LOS (coverage)	0.38	0.00–0.88
Gout patients with colchicine or NSAID use (%)	9.25	
Episodes before ULT episodes	1	0–3
Episodes during ULT episodes	1	1–3
Episodes after ULT episodes	0	0–2
Time on colchicine or NSAIDs/LOS (coverage)	0.11	0.03–0.49

LOS: length of stay; ULT: urate-lowering therapy.

**Figure 1.**
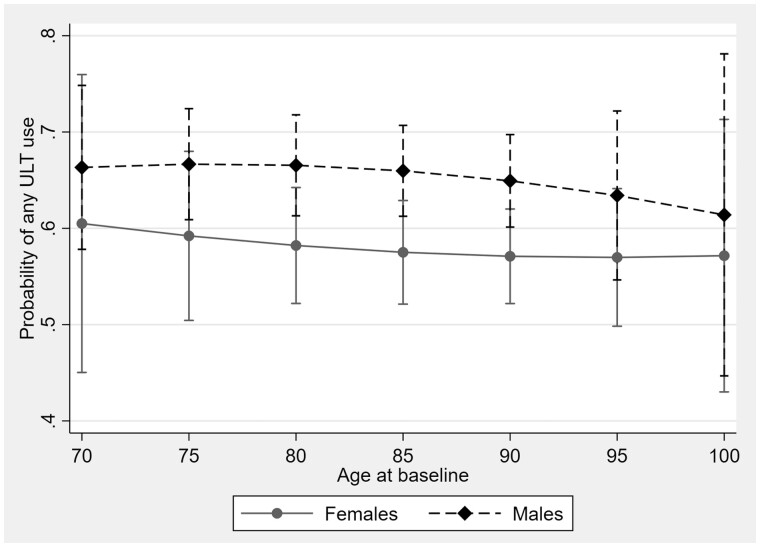
Urate-lowering therapy use in residents with gout: proportion on a urate-lowering therapy. ULT: urate-lowering therapy

A higher proportion of residents who received ULT were male (56%), and ULT use tended to be lower at older ages, although these differences were not statistically significant across age groups (Fig. 1).

**Figure 2.**
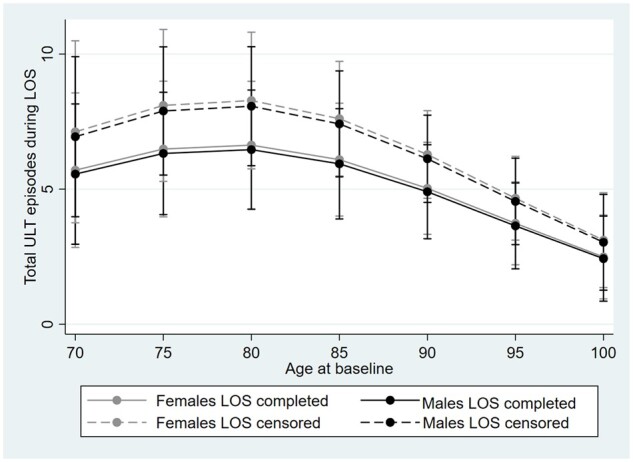
Total number of urate-lowering therapy episodes during length of stay in residents with gout who had a complete or incomplete/censored (i.e. resident came in before/after data collection period) length of stay during the study period. LOS: length of stay; ULT: urate-lowering therapy

Fig. 2 displays the total number of ULT episodes of residents with gout by sex. The number of ULT episodes was higher in those residents who had censored stays in the facility. The total number of ULT episodes declined with increasing age >80 years in all groups and for both sexes.

**Figure 3.**
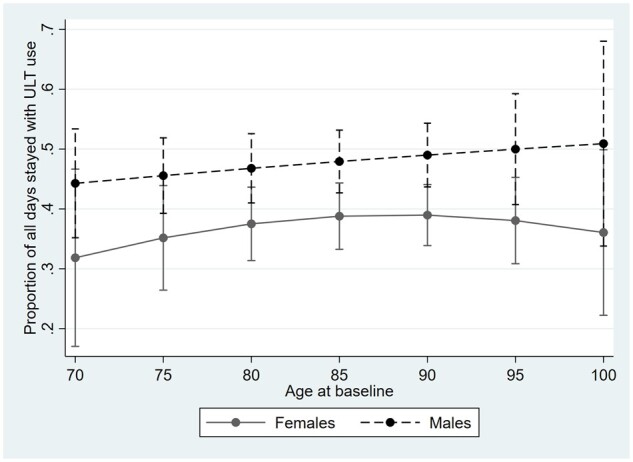
Proportion of length of stay associated with use of urate-lowering therapy. ULT: urate-lowering therapy

Male residents with gout tended to be on ULT for a greater proportion (median = 0.55, range = 0–1) of their stay in aged care facilities, compared with female residents with gout (median = 0.18, range = 0–1; Fig. 3). In males, this proportion increased with age, a trend also seen in females until 90 years of age, when a decrease occurred.

Gout management guidelines recommend that if a patient has had two gout flares, they should be on ULT. Of those residents with gout, 9.25% received colchicine or an NSAID at some point during their stay (Table 2). Use of colchicine/NSAID (i.e. a proxy measure for gout flares) before, during and after ULT episodes had a median of one episode (range = 0–3), one (range = 1–3) and zero (range = 0–2) respectively, and residents with gout received colchicine/NSAIDs for a median of 11% of their LOS (range = 3–49%). It was found that 80 (33%), 109 (45%) and 52 (22%) residents with gout received colchicine/an NSAID before, during and after ULT episodes, respectively (Table 2).

Table 3 shows that the probability of using a ULT was significantly greater for males, with no significant associations for specific metabolic co-morbidities associated with gout [27].

**Table 3.** Marginal effects for probability of any urate-lowering therapy use

Variable	Estimate (s.e.)	95% CI	*P*-value
Age at baseline, decades	0.02 (0.02)	−0.01, 0.05	0.19
Male	0.11 (0.02)	0.06, 0.16	0.00
Co-morbidities			
Hypertension	−0.01 (0.03)	−0.07, 0.04	0.64
Renal disease	0.04 (0.03)	−0.01, 0.10	0.10
Dementia	0.00 (0.02)	−0.05, 0.04	0.90
Diabetes	0.00 (0.03)	−0.05, 0.05	0.92
Heart disease	−0.01 (0.03)	−0.07, 0.05	0.70
Length of stay, decades	0.08 (0.05)	−0.01, 0.17	0.08
Facility urban/rural category			
Inner regional Australia	−0.06 (0.03)	−0.12, 0.00	0.05
Outer regional Australia	0.06 (0.08)	−0.10, 0.22	0.47

Reference groups are: female; and major cities of Australia. Generalized estimating equations regression was performed, with the outcome of ULT use in a given year. Marginal effects are interpretable as the change in the prevalence of ULT use associated with a change in a given independent variable from the base/reference level.

## Discussion

Pharmacological management of gout in this RACF cohort showed some indications of concordance with guidelines. Gout guidelines state that a patient who has had two gout flares in a 12-month period should be on life-long ULT to keep their urate levels consistently <0.36 mmol/l to prevent gout flares. In this study, 62% of residents with a gout diagnosis received a ULT, which is higher than reported for people in the community (ranges from 10 to 46%) [28, 29]. However, residents had a median of one episode of ULT that was of a very short duration (median = 4 days). This result indicates that most residents with gout were at high risk of recurrent flares and, ultimately, joint and possibly organ damage [30]. A short duration of ULT use and a single dispensing of ULT are also problems in the community setting [28, 31]. There are several potential reasons for this short duration of ULT. Upon initiation of ULT, gout flares are well described. ULT is commonly but inappropriately ceased. Also, given the multimorbidity experienced by this cohort, hospitalizations, serious illness and death are common. These occurrences would result in ceasing of medications, as evidenced by 49% of this population dying during the study period. The median total number of ULT treatments during the study period was 52 days, which indicates that residents are prescribed multiple short ULT courses throughout their RACF stay. Recurrent ULT stopping and restarting is likely to lead to further flares [1]. These patterns of ULT use are discordant with evidence-based gout management guidelines.

Once a patient is commenced on ULT, they should remain on ULT. This would mean that residents’ medication records should ideally reveal one ULT episode for the duration of their stay in the RACF. Our results showed that the total number of ULT episodes declined with increasing age >80 years old in both males and females, but with an increase in ULT use as a proportion of their LOS observed only in males. This suggests differences in gout treatment based on sex, whereby males received more concordant gout management compared with female residents. We identified differences in the likelihood of receiving ULT and in the time spent taking ULT between males and females. Males were more likely to be on a ULT across all age groups and for a longer portion of their LOS in the facility. These sex differences might be driven by patient factors, the prescriber, or both. Further research is required to explore whether management of gout is sex dependent in various settings and whether greater vigilance is required for management of female residents with gout [32, 33].

In this study, we used the administration of colchicine or an NSAID as a proxy for a gout flare in residents with gout. According to the guidelines, two episodes of colchicine/NSAID should occur within 1 year before the first episode of ULT. We found that ∼9% of residents with a gout diagnosis in their record were administered colchicine or an NSAID. These few episodes of use of colchicine/NSAID could suggest that residents experienced infrequent gout flares. However, we suspect that the few episodes of colchicine/NSAID use, coupled with relatively few and short durations of ULT episodes, are instead indicative of gout management discordant with guidelines. In fact, of those who received colchicine/NSAID, 33% (80 of 241) received it before their initial ULT administration. Among residents who received colchicine/NSAID before their first ULT, most had only one episode of colchicine/NSAID, although gout guidelines state that patients should have two gout flares before ULTs are considered. However, we do not know which medications these residents had received before admission, and based on the mean age of gout onset generally (60 years old [34]), it is likely that many of these residents had used ULTs, NSAIDs or colchicine before admission, because the median age at RACF admission is 85 years for males and 87 years for females. A recent study indicated that patients with gout living in the community and receiving prophylactic medications, such as colchicine, upon ULT initiation is low and that discontinuation of ULTs in the next 12 months is high [35]. Interestingly, in our study a larger portion of residents received colchicine/NSAID during (*n* = 109) or after (*n* = 52) their first episode of ULT. These data suggest use concordant with guidelines, which state that initial administration of ULT should be co-prescribed with prophylactic colchicine/NSAID to circumvent the increased risk of flares during ULT initiation [36]. However, the pattern of colchicine/NSAID use during ULT episodes that we found is more likely to be explained by residents receiving an inadequate dose of ULT to lower urate sufficiently. Of note, in older adults, colchicine is strongly preferred, given the increased prevalence of co-morbidities such as hypertension, heart failure and renal impairment, which can be exacerbated with NSAIDs but not colchicine [37].

Limitations of this study include the data being sourced from a single aged care provider that might not be representative nationally. However, the data are demographically representative of people who use residential aged care in New South Wales and are similar to the national population of Australians who use residential aged care [22]. We did not have medication use data before residents entered the facility, hence it was not possible to ascertain whether the resident had been treated with ULT before admission. This would also be useful to interpret colchicine/NSAID use before ULT, for example. It is likely that most of the residents with a diagnosis of gout had been treated for the condition before RACF admission because the average age at gout diagnosis in the community is substantially younger than the age at RACF admission [34]. The use of NSAIDs as a proxy for gout flares in this study might result in misclassification of a gout flare. This is because we did not have data regarding indications for medication administration. However, to minimize this, we ensured that all residents in the cohort had a clinical diagnosis of gout (through electronic health record, ACFI or ULT administration). Together with colchicine, NSAID use is considered the first line for gout flares over CSs [24] and is also used very commonly by patients with gout in the community as an accessible, over-the-counter treatment for gout, a behaviour that is also likely to continue in RACFs [37]. Another limitation is that we did not have access to pathology data to determine whether urate levels were adequately controlled with the ULT. Future studies should examine how ULT use changes as people transition from having their gout treated in the community and move into residential aged care. Furthermore, we did not have access to data on symptoms, severity of the resident’s gout or side effects of medications, which can influence treatment decisions, although allopurinol is very well tolerated. Likewise, information regarding initiating and stopping ULT would be very useful. Performance of sensitivity analyses using varying durations of gaps between episodes could determine whether shorter or longer gaps between episodes affect adherence to management guidelines.

The study strengths include the establishment of a new method for assessing disease management in RACFs that maximizes the use of existing electronic data, instead of relying on time-intensive chart reviews. Furthermore, we maximized the use of all available electronic datasets held by the aged care provider, including medication administration data, ACFI data and clinical notes in the electronic health record, to identify gout, co-morbidities and gout-specific medications. Co-morbidities and other factors were also examined and controlled for in the analyses. Several studies exploring the prevalence of various conditions and longitudinal medication use in residential aged care, using these data and similar methods, have been published [6, 22, 38–40].

### Conclusions

RACFs are an ideal setting for optimal medication adherence, given that they are a controlled environment, in which residents are given their prescribed medications daily and under supervision. In this setting, routinely collected electronic records of medication use and health conditions can be used to compare real-world management of conditions with guideline recommendations. Disorders with relatively standard treatment guidelines together with medications that are highly specific to the condition, such as gout, are model candidates for this purpose. This is the first study to use electronic medication administration data from RACFs to examine the quality of gout management in residents. This study showed that gout, a condition which has notoriously poor medication adherence in the community, also has suboptimal management within the RACF setting. Many residents with gout were either not receiving preventative ULTs or were receiving them in non-concordant ways, such as for short periods of time rather than continuously. The electronic systems used by RACFs can be used to improve gout management in RACFs, for example by prompting prescribers to treat gout according to guidelines, such as through flagging residents with gout where ULTs are indicated and through reminders that if ULTs are indicated, they should not be stopped.

## Supplementary Material

rkac091_Supplementary_Data

## Data Availability

The data underlying this article will be shared on reasonable request to the corresponding author.
