# Supplementary material for: Measuring quality of gout management in residential aged care facilities
Source: Rheumatol Adv Pract. 2022 Nov 28;6(3):rkac091. doi: 10.1093/rap/rkac091 (PMC9710438; doi:10.1093/rap/rkac091)
Supplement: rkac091_Supplementary_Data [file rkac091_supplementary_data.docx]

**Supplementary Table S1.** Summary of Australian/New Zealand pharmacological gout management guidelines^21^

| **Area of management** | **Recommendations** |
| --- | --- |
| Acute gout management | - Low dose colchicine (total of 1.5 mg/day for 1 day only) - NSAIDs - Glucocorticoids - Drug selection is influenced by comorbidities and concomitant medications - Combination therapy may be appropriate |
| Target serum urate levels | - Target SUA of <0.36 mmol/l - Target SUA of <0.3 mmol/l for tophaceous gout - Target SUA should be maintained in the long term and when reached should be monitored every 3-6 months |
| Urate-lowering therapy | - ULT dosage should be adjusted until target SUA is achieved - Allopurinol is first-line ULT   - During acute attacks, allopurinol should not be stopped - If allopurinol is not tolerated or cannot achieve target SUA, second-line ULT options are:   - Probenecid, benzbromarone* or febuxostat   - Choice of second-line ULT depends on patient comorbidities and/or concomitant medications |
| Prophylaxis when starting ULT | - Low-dose colchicine (0.5-1.0 mg/day for 6 months) - NSAIDs - Low-dose glucocorticoids |
| Co-morbidities and related medications | - *Impaired renal function:* tolerance to allopurinol takes preference over renal function when escalating dosage from a low dose (maximum dose of 900 mg/day) - *Impaired renal function, gastrointestinal disease or diabetes:* careful use of NSAIDs and glucocorticoids - *Renal impairment and strong CYP3A4 inhibitors*: reduce colchicine dose - *Azathioprine:* if co-prescribed with allopurinol or febuxostat, azathioprine dosage should be reduced and carefully monitored - *Nephrolithiasis:* uricosurics should be used with caution |
| Tophi | - Tophi are a definite indication for intensive ULT, although surgery is not indicated in most cases - Short-term treatment of pegylated uricase recommended for refractory cases of tophi |

Abbreviations: NSAID, non-steroidal anti-inflammatory drug; SUA, serum urate; ULT, urate lowering therapy; CYP3A4; Cytochrome P450 3A4

*benzbromarone not registered in Australia.
